# Mechanism of efficacy of trabectedin against myxoid liposarcoma entails detachment of the FUS-DDIT3 transcription factor from its DNA binding sites

**DOI:** 10.1186/s13046-024-03228-z

**Published:** 2024-11-26

**Authors:** Ilaria Craparotta, Laura Mannarino, Riccardo Zadro, Sara Ballabio, Sergio Marchini, Giulio Pavesi, Marta Russo, Salvatore Lorenzo Renne, Marina Meroni, Marianna Ponzo, Ezia Bello, Roberta Sanfilippo, Paolo G. Casali, Maurizio D’Incalci, Roberta Frapolli

**Affiliations:** 1 Department of Experimental Oncology, Istituto di Ricerche Farmacologiche Mario Negri IRCCS, Milan, Italy; 2 Department of Biomedical Sciences, Humanitas University, Via Rita Levi Montalcini 4, Milan, Pieve Emanuele 20072 Italy; 3 Laboratory of Cancer Pharmacology, IRCCS Humanitas Research Hospital, Via Manzoni 56, Milan, Rozzano 20089 Italy; 4 SC Patologia Clinica, SS Laboratorio Genetica Medica, Fondazione IRCCS Ca’ Granda Ospedale Maggiore Policlinico, Milan, Italy; 5 Dipartimento Di Bioscienze, Università Degli Studi Di Milano, Milan, 20133 Italy; 6 Department of Experimental Oncology, European Institute of Oncology (IEO) IRCCS, Milan, 20139 Italy; 7 Anatomic Pathology Unit, IRCCS Humanitas Research Hospital, Via Manzoni 56, Milan, Rozzano 20089 Italy; 8 Adult Mesenchymal Tumour Medical Oncology Unit, Fondazione IRCCS Istituto Nazionale Dei Tumori, Via Venezian 1, Milan, 20133 Italy

**Keywords:** Trabectedin, Liposarcoma, Myxoid, Chromatin immunoprecipitation sequencing, Adipogenesis, Heterografts, Recombinant fusion proteins

## Abstract

**Background:**

The marine drug trabectedin has shown unusual effectiveness in the treatment of myxoid liposarcoma (MLPS), a liposarcoma characterized by the expression of the FUS-DDIT3 chimera. Trabectedin elicits a significant transcriptional response in MLPS resulting in cellular depletion and reactivation of adipogenesis. However, the role of the chimeric protein in the mechanism of action of the drug is not entirely understood.

**Methods:**

FUS-DDIT3-specific binding sites were assessed through Chromatin Immunoprecipitation Sequencing (ChIP-Seq). Trabectedin-induced effects were studied on pre-established patient-derived xenograft models of MLPS, one sensitive to (ML017) and one resistant against (ML017ET) trabectedin at different time points (24 and 72 h, 15 days). Data were integrated with RNA-Seq from the same models.

**Results:**

Through ChIP-Seq, here we demonstrate that trabectedin inhibits the binding of FUS-DDIT3 to its target genes, restoring adipocyte differentiation in a patient-derived xenograft model of MLPS sensitive to trabectedin. In addition, complementary RNA-Seq data on the same model demonstrates a two-phase effect of trabectedin, characterized by an initial FUS-DDIT3-independent cytotoxicity, followed by a transcriptionally active pro-differentiation phase due to the long-lasting detachment of the chimera from the DNA. Interestingly, in a trabectedin-resistant MLPS model, the effect of trabectedin on FUS-DDIT3 rapidly decreased over time, and prolonged treatment was no longer able to induce any transcription or post-transcriptional modifications.

**Conclusions:**

These findings explain the unusual mechanism underlying trabectedin's effectiveness against MLPS by pinpointing the chimera's role in inducing the differentiation block responsible for MLPS pathogenesis. Additionally, the findings hint at a potential mechanism of resistance acquired in vivo.

**Graphical Abstract:**

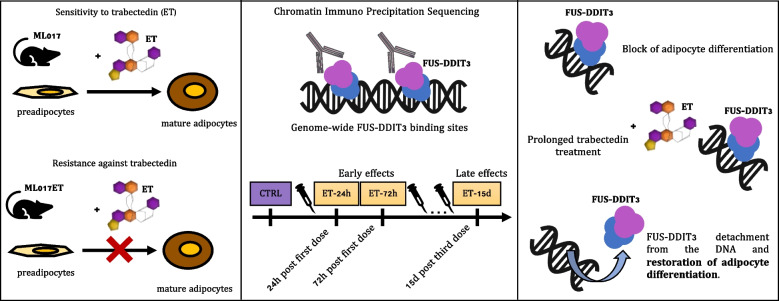

**Supplementary Information:**

The online version contains supplementary material available at 10.1186/s13046-024-03228-z.

## Background

Trabectedin is a marine drug known to have a pleiotropic mechanism of action. It binds to the minor groove of the DNA affecting transcription and leading to cytotoxic, anti-angiogenic, and immunomodulatory effects [1, 2]. It was approved in 2007 by the EMA (European Medicines Agency) for the treatment of soft-tissue sarcomas (STS) [3]. In particular, trabectedin received also FDA (Food and Drug Administration) approval in 2015 for the treatment of liposarcoma and leiomyosarcoma which seem to be more sensitive to trabectedin than other sarcomas [4]. Myxoid liposarcoma (MLPS) is a subtype of liposarcoma. It is characterized by the expression of the FUS-DDIT3 chimeric protein caused by the chromosomal translocation t(12;16)(q13;p11) considered the pathogenic event leading to MLPS development [5–9]. The DNA binding domain of the chimeric protein is located on DDIT3. This gene is member of the CCAAT/enhancer-binding protein (C/EBP) family of transcription factors. Its protein product acts as a dominant-inhibitor by forming heterodimers with other C/EBP members [10]. However, when involved in the fusion protein, the effect is a direct downregulation of the expression of the transcription factors c/EBPa and PPARg, the master regulators of adipogenesis, by blocking the late stages of adipogenesis with the consequent accumulation of immature adipoblasts that continue to proliferate [11, 12]. MLPS is characterized by a higher chemo- and radio-sensitivity than other adult-type STS. Radiotherapy or chemotherapy is frequently used in neoadjuvant settings to achieve tumor shrinkage and help obtain adequate surgical margins. Anthracycline-based chemotherapy regimen is the standard-of-care first-line systemic treatment for advanced MLPS. However, because of its unusual activity in this tumor histotype, trabectedin may represent an effective option for first-line therapy when anthracyclines cannot be prescribed [13]. Previous data demonstrate that in MLPS trabectedin allows the reactivation of the adipogenic process overcoming the effect of the chimeric protein [14]. The molecular features involved in the response of MLPS to trabectedin were studied in MLPS patient-derived xenografts (PDXs) sensitive to or resistant against trabectedin integrating genomic and transcriptomic data. In the PDX-sensitive ML017 model, a two-phase effect was observed with an early cytotoxic response, where the activated pathways are those mainly involved in the transcriptional regulation of *TP53* and transcription, followed by a later differentiation phase. This differentiation phase corresponds to a phenotypic modification of the neoplastic tissue (e.g. extracellular matrix organization, collagen production) similar to what is observed in the patient’s specimen after therapy [15, 16]. Intriguingly, the adipocyte differentiation was not observed in the ML017ET, the trabectedin-resistant PDX model. This model was derived through repeated in vivo treatments that led to the loss of three chromosomic regions (i.e. 4p15.2, 4p16.3, and 17q21.31) upon drug pressure with the loss of expression of the *UVSSA* gene involved in the transcription-coupled nucleotide excision repair (TC-NER) pathway. This repair mechanism is crucial for the cytotoxic activity of trabectedin [17]. In this model, trabectedin induced a strong transcriptional response at an early time point (i.e. 24 h) which was quickly extinguished with minimal consequences on tumor morphology and growth [15, 16].

Although these results highlight the ability of trabectedin to modulate transcription and restore adipogenesis, the role of the FUS-DDIT3 chimera in this process is unknown. The direct effect of trabectedin treatment on genome-wide DNA binding of the chimera has not been investigated so far [18]. The availability of these precious preclinical models with differential sensitivities to trabectedin allowed us to study the FUS-DDIT3-mediated molecular mechanisms that guide the response to the drug, using a Chromatin Immunoprecipitation Sequencing (ChIP-Seq) approach. The main objectives of the study were: 1. to assess the FUS-DDIT3 genomic distribution in untreated MLPS growing in vivo (CTRL); 2. to discover binding pattern differences in genomic profiles of FUS-DDIT3 between a trabectedin-responsive model and a resistant one; 3. to assess the modulation of transcription induced by trabectedin at various time points following both single and repeated treatments.

## Methods

### Animals

All the procedures involving animals and their care were conducted in conformity with the following laws, regulations and policies governing the care and use of laboratory animals: Italian Governing Law (D.lgs 26/2014; Authorization n.19/2008-A issued March 6, 2008, by the Ministry of Health), Mario Negri Institutional Regulations and Policies providing internal authorization for persons conducting animal experiments (Quality Management System Certificate—UNI EN ISO 9001:2008—Reg. No. 6121), the NIH Guide for the Care and Use of Laboratory Animals (2011 edition) and EU directives and guidelines (EEC Council Directive 2010/63/UE) and guidelines for the welfare and use of animals in cancer research [19].

Six- to eight-week-old female CD1 nude mice, purchased from Charles River Laboratories (Calco, Italy), were housed in individually ventilated cages, with sterilized food and water ad libitum and handled under specific pathogen-free conditions in the Animal Care Facility of Mario Negri Institute, which meets international standards for animal welfare. Mice were regularly checked by a certified veterinarian who is responsible for health monitoring, animal welfare supervision, experimental protocols, and review of procedures.

### Drugs

Trabectedin, kindly supplied by PharmaMar S.A., was dissolved in water at a stock concentration of 0.05 mg/ml and further diluted to 0.015 mg/ml in saline immediately before use.

### Tumor models

Myxoid liposarcoma patient-derived xenograft (PDX) models were obtained as previously described [16, 20]. Briefly, human tumor biopsies were cut into small fragments of about 3 × 3 mm and subcutaneously (s.c.) engrafted in female athymic nude mice under isoflurane anesthesia. The histological features of the tumors were verified after each passage in mice and compared to the original human sample in order to maintain the clinical relevance of the model. When tumor mass reached about 400 mg, ML017, and ML017/ET tumor-bearing mice were treated with trabectedin 0.15 mg/kg every 7 days for three times (q7dx3). They were sacrificed 24 (ET-24h) and 72 (ET-72h) hours after the first dose and 15 days (ET-15d) after the third and last dose of treatment. Mice used as controls were treated with saline solution. Tumor growth was measured using Vernier caliper and tumor volume was approximated by the formula: $\frac{length\times {width}^{2}}{2}$.

### Chromatin immunoprecipitation (ChIP) and ChIP-sequencing (ChIP-Seq)

When animals were sacrificed, tumors were fragmented with the use of a scalpel to break up the tissue and immediately treated with 1% formaldehyde for 10 min at room temperature (RT). Then, the reaction was quenched by adding glycine 0.125 M. After spinning and washing with PBS solution, the cross-linked tissue samples were stored at -80 °C. During ChIP experiments, lysis buffer 1 (50 mM Hepes–KOH, 140 mM NaCl, 1 mM EDTA, 10% glycerol, 0.5% NP-40, 0.25% Triton X-100, ddH_2_O), supplemented with protease inhibitors (Roche, Basel, Switzerland) was added to previously fixed-samples; they were homogenized using an ultra-turrax (VWR, Radnor, Pennsylvania, USA). After, samples were washed in lysis buffer 2 (10 mM Tris–HCl, 200 mM NaCl, 1 mM EDTA, 0.5 mM EGTA, ddH_2_O) and lysis buffer 3 (10 mM Tris–HCl, 100 mM NaCl, 1 mM EDTA, 0.5 mM EGTA, 0.1% Na-deoxycholate, 0.5% N-Lauroylsarcosine, ddH_2_O), both supplemented with protease inhibitors (Roche, Basilea, Switzerland). Obtained chromatin was sheared on Bioruptor sonicator (Diagenode) set at high potency for 30 pulses, each one comprised of 60 s ON and 30 s OFF. After checking the chromatin smear, 1% of the sample volume was collected and stored at -20 °C to be next used as input sample, while 10 µg of antibody (DDIT3, Proteintech), previously incubated all day at 4 °C in a rotation wheel with Dynabeads (Life Technologies, Carlsbad, California, USA), was added to the remaining sample volume. After overnight incubation in a rotation wheel at 4 °C, immunoprecipitated samples (IPs) were washed with RIPA wash buffer (50 mM Hepes–KOH, 500 mM LiCl, 1 mM EDTA, 1% NP-40, 0.7% Na-deoxycholate, ddH_2_O) for 6 times; after spinning, dynabeads were removed in a magnetic stand, samples were eluted in Elution Buffer (TE 1X and 2% SDS) and incubated overnight at 65 °C to remove crosslinks. The recovered material was purified using QIAquick PCR purification kit (Qiagen, Hilden, Germania) and DNA was quantified using Qubit® dsDNA High Sensitivity Assay Kit (Life Technologies, Carlsbad, California, USA). As negative control of the ChIP protocol, an IP reaction against IgG (Cell Signaling, Danvers, Massachusetts, Stati Uniti) was performed.

ChIP-Seq libraries were created adapting the TruSeq ChIP protocol (Illumina, San Diego, California, USA): around 50 ng of IPs and input were used for library preparation. Libraries were run on a NextSeq 500 sequencer (Illumina, San Diego, California, USA) using a 1 × 75 bp high-output kit (Illumina, San Diego, California, USA) with 8 IPs (50 M reads per sample) or 4 input samples (100 M read per sample) for run.

### Data analysis

#### Data pre-processing

Raw sequences were demultiplexed with *bcl2fastq* conversion software (Illumina, San Diego, California, USA), and the quality control of *fastq* files was done with FastQC [21]. Raw sequences were processed with *bcbio-nextgen* [22] pipeline configured as follows: read mapping was done with Bowtie2 v2.2.5 [23] against hg19 UCSC human genome with the preset option “very sensitive”, that consists in the following parameters: -D 20 (seed extension) -R 3 (number of re-seed reads) -N 0 (number of allowed mismatches) -L 20 (length of the seed substring) -i S,1,0.50 (to control the intervals between seed substrings), resulting in a much slower, but more accurate alignment. A set of “grey regions”, i.e. regions of the input sample with abnormally high read coverage in both IP and input, was calculated for each pair sample/control pair with the Python implementation of the R package GreyListChIP [24]; MACS2 v.2.2.7.1 [25] was used for peak calling with default parameters and a q-value threshold of 0.05.

#### Motif analysis

Motif analysis of peaks derived from PDX under untreated conditions was performed with PScanChIP [26] using the Jaspar 2018 NR [27] database as a motif descriptor database.

#### Consensus of ChIP-Seq peaks

For PDX experiments for which at least three replicates were available at basal conditions, the final peak consensus set was calculated based on overlapping regions through the *dba.peakset* function of the DiffBind package [28]. A stringent rule was applied, keeping only peaks that were identified in all replicates.

#### Differentially bound peaks analysis

Regions of differential binding (i.e. differentially bound peaks, DBPs) between two conditions were identified with the DiffBind R package v.3.4.1 [28]. First, a consensus set of peaks for each treatment was created, including only peaks present in at least three replicates. DiffBind implementation of DESeq2 [29] was then used to identify DBPs in each comparison.

#### DBPs annotation

DBPs were annotated with the R package ChIPSeeker v.1.30.0 [30]. The promoter region was defined as 5 kb upstream and 1 kb downstream of the transcription start site (TSS) of genes, while distal intergenic regions were defined at a distance of up to 1000 kb upstream of the promoter.

#### Enrichment analysis

Pathway enrichment analysis was performed with clusterprofiler v.3.18.1 [31] with both Reactome [32] and Wikipathways [33] databases, with and adjusted *p*-value cut-off of 0.05.

#### Enrichment map

Enrichment maps was drawn using the EnrichmentMap application of Cytoscape [34]. Edges were drawn with an overlap of at least 0.5, defined as the size of the intersection of two sets of genes divided by the size of the smaller set.

#### Data integration with RNA-seq

Integration of ChIP-Seq and RNA-Seq data was done at both the gene and the pathway level. RNA-Seq data were retrieved from Mannarino, Craparotta et al*.* [15] (EGAS00001004901), in which the same PDX models were analyzed with the same experimental design.

## Results

### Establishment of ad-hoc experimental design to study FUS-DDIT3 binding activity

To characterize for the first time the DNA binding pattern of the FUS-DDIT3 oncoprotein in myxoid liposarcoma (MLPS) tumor models, chromatin immunoprecipitation followed by sequencing assay (ChIP-Seq) was performed on ex vivo xenograft models using an antibody against the DDIT3 transcription factor. Experiments were performed as previously described [15] on two patient-derived xenograft (PDX) models of MLPS: ML017, and ML017ET, sensitive to and resistant against trabectedin, respectively [16, 20]. The whole list of samples and associated metadata are reported in Table S1.

### FUS-DDIT3 binding pattern in ET-sensitive and -resistant PDX models of MLPS

We initially aimed to characterize the DNA binding sites of the FUS-DDIT3 chimeric protein and the potential differences between ML017 and ML017ET models. To this end, ChIP experiments were performed with an antibody against the transcription factor encoded by the *DDIT3* gene whose endogenous form is not expressed in our PDX models as assessed by western blot analysis (data not shown). We initially investigated the effectiveness of the anti-DDIT3 antibody to select DNA binding sites associated with the FUS-DDIT3 protein. Analysis of the consensus peaks derived from the ML017 model (see Materials and Methods) gave 19,966 peaks, that were analyzed through motif analysis for the identification of the most enriched transcription factor binding sites (Table S2). We found that the DDIT3 motif stored in the Jaspar database [27] was the most significantly enriched in the peak regions, confirming the effectiveness of our antibody and the consequent robustness of the results (Figure S1, Table S3).

**Fig. 1:**
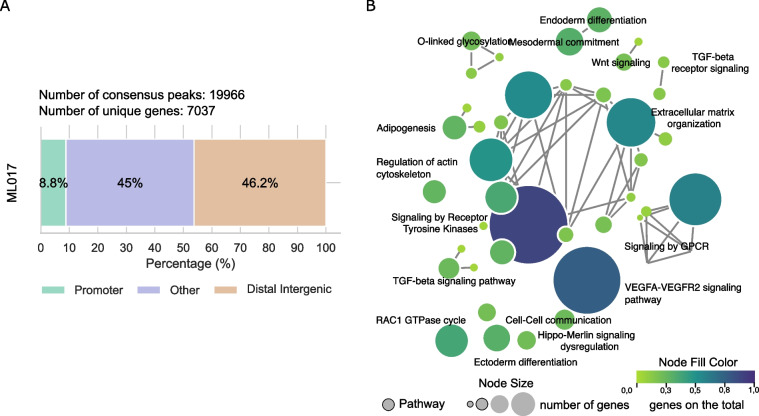
FUS-DDIT3 peaks annotation in ML017 controls. **A** Genomic distribution (%) of the annotated peaks from the consensus of the control (CTRL) condition in ML017. Color regions as reported in the legend. **B** Enrichment map obtained from the annotated genes of the consensus CTRL of ML017. Pathways are reported as circles, they are connected when sharing at least 50% of the genes. The node size is proportional to the number of genes in the pathway. The node fill color is proportional to the fraction of the significant genes over the total number of genes in the pathway (see Materials and Methods)

We wondered which genes were potentially related to the FUS-DDIT3 peaks and whether they were connected to specific biological pathways. To find this out, we annotated the 19,966 consensus peaks related to FUS-DDIT3 binding. They resulted in 7037 unique genes associated with them (Table S2). Almost 9% of these peaks were located in promoter regions, while the remaining 91% were in introns, exons, or UTR regions (defined as “other”), or locations further than 1000 kb from known transcription start sites (TSS) (Fig. 1A). A total of 53 pathways were found to be enriched in the putative target genes (Table S4). The pathways with the highest number of genes involved (> 100) were those regulated by the *GPCR* gene*,* signaling by receptor tyrosine kinase, VEGFA pathway, and extracellular matrix organization. To resolve this complexity, we computed an enrichment map in which different pathways sharing more than 50% of the genes were connected resulting in a network which encompasses the main represented biological processes (Fig. 1B). In addition to the identified pathways, the vast majority of genes regulated by FUS-DDIT3 chimera at baseline were involved in adipogenesis, white fat cells differentiation, Hippo-Merlin and Wnt signaling, TGF-beta and VEGFA-VEGF2 signaling pathways, extracellular matrix organization, cell–cell communication, and the differentiation of the ectoderm and endoderm. This finding is consistent with the hypothesis that the transcriptional programs that control adipogenesis and differentiation processes in MLPS are directly regulated by FUS-DDIT3.

We finally investigated whether the mechanism of acquired resistance against trabectedin can be explained at the molecular level by an impairment or a different DNA binding pattern of the FUS-DDIT3 chimera. To that end, we compared the ChIP-Seq profiles of the untreated conditions of the ML017ET model to those of the ML017. We found only 19 differentially bound peaks (DBPs), *i.e.* enriched regions present in only one of the two conditions or with a significant difference in enrichment, corresponding to 18 unique target genes (Figure S2, Table S5) showing a highly overlapping binding pattern of FUS-DDIT3 between the control ML017 and ML017ET. This result suggests that the mechanism leading to acquired resistance against trabectedin is not driven by a different pattern of FUS-DDIT3 DNA binding.

### FUS-DDIT3 binding is modulated by prolonged treatment with trabectedin in MLPS trabectedin-responsive models

We have previously shown that in MLPS models trabectedin can restore adipocyte differentiation after prolonged treatment [15]. Thus, we explored whether, from a mechanistic point of view, the reactivation of adipocyte differentiation is directly driven by the loss of the FUS-DDIT3 chimera. To this aim, we performed an additional ChIP-Seq experiment in the ML017 model treated with trabectedin under the same treatment scheme used in our previous work [15] and summarized in Figure S3.

**Fig. 2:**
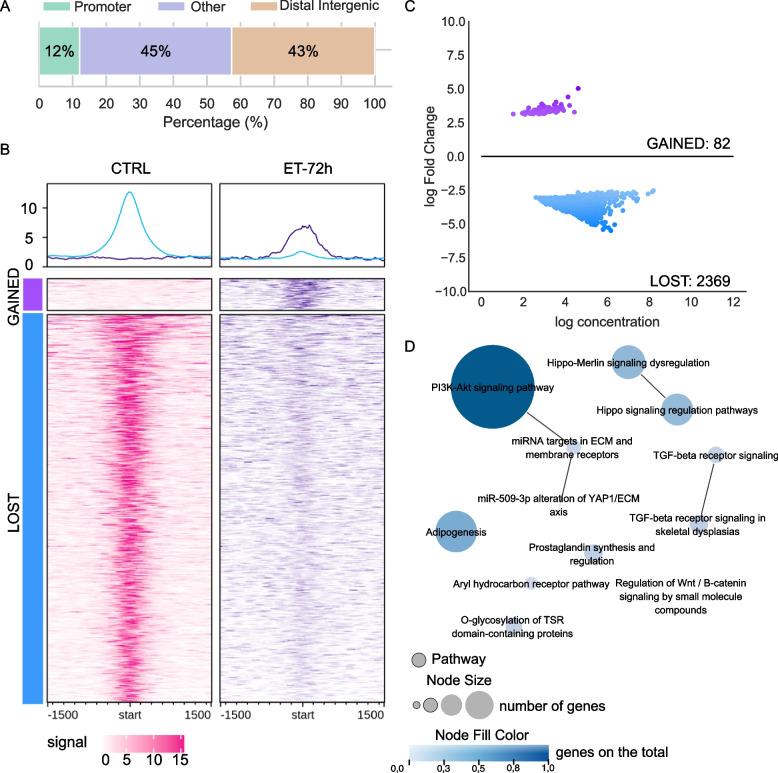
Differential binding in ET-72h versus CTRL in ML017. **A** Genomic distribution (%) of the annotated peaks from the DBPs of the comparison ET-72h *versus* CTRL in ML017. Color regions as reported in the legend. **B** Read enrichment analysis around DBPs in CTRL and ET-72h conditions in ML017. **C** MAplot of log concentration versus log Fold Change of the identified DBPs. **D** Enrichment map of the annotated genes associated with pathways. Pathways are reported as circles, they are connected when sharing at least 50% of the genes. The node size is proportional to the number of genes in the pathway. The node fill color is proportional to the fraction of the significant genes over the total number of genes in the pathway (see Materials and Methods)

**Fig. 3:**
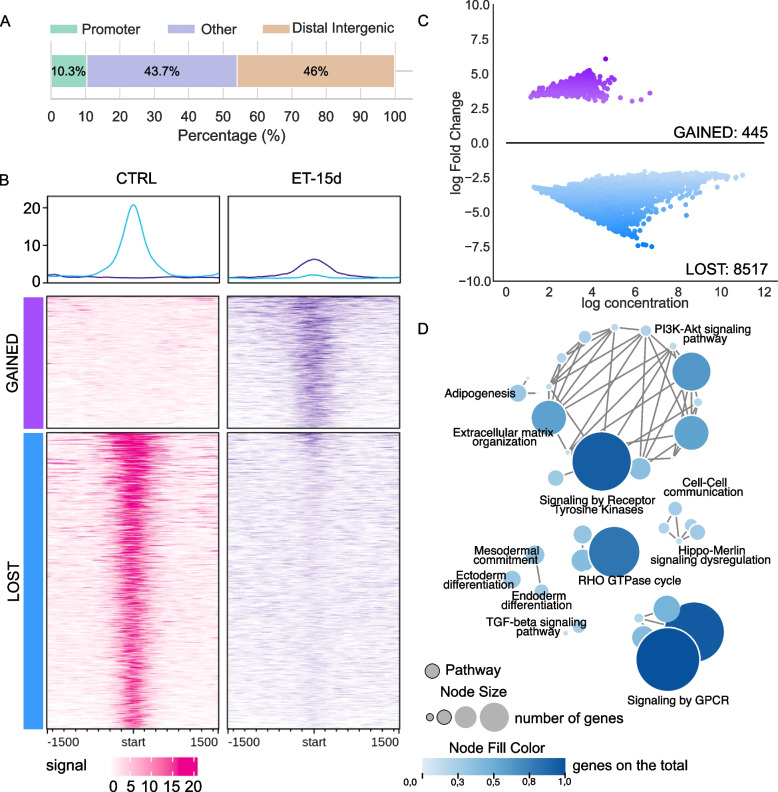
Differential binding in ET-15d versus CTRL in ML017. **A** Genomic distribution (%) of the annotated peaks from the DBPs of the comparison ET-15d *versus* CTRL in ML017. Color regions as reported in the legend. **B** Read enrichment analysis around DBPs in CTRL and ET-15d conditions in ML017. **C** MAplot of log concentration versus log Fold Change of the identified DBPs. **D** Enrichment map of the annotated genes associated with pathways. Pathways are reported as circles, they are connected when sharing at least 50% of the genes. The node size is proportional to the number of genes in the pathway. The node fill color is proportional to the fraction of the significant genes over the total number of genes in the pathway (see Materials and Methods)

First, we compared treated samples against the CTRL condition (Table S6). As shown in Figure S4, the number of DBPs increases along with the time of treatment, from 41 to 8962, following the same trend as the DEGs identified with the transcriptional analysis [15]. This may suggest a possible correlation between FUS-DDIT3 binding activity and the transcriptional modulation induced by trabectedin. This was investigated further under the ET-72h and the ET-15d conditions. As shown in Fig. 2A, the 2451 DBPs at 72 h after the first dose mapped preferentially far from promoter regions. Of these, only 82 (3.35%) were newly acquired bound peaks (defined as “GAINED”) and were associated with genes with no biological relevance. Otherwise, the remaining 2369 (96.65%) DBPs were no longer bound or bound with a weaker binding potential (defined as “LOST”) in the ET-72h (Fig. 2B and C). These peaks were annotated to 1830 unique genes which were involved in 12 significant biological functions (Fig. 2D). Interestingly among these, we found most of the pathways that were under the control of the FUS-DDIT3 fusion protein at the basal level, like adipogenesis, and the Wnt, TGF-beta, and PIK3CA pathways. This finding suggests that at the molecular level trabectedin seems to displace the chimera from its canonical binding sites. When we analyzed the ET-15d experimental condition the 8962 DBPs were mainly located in regions outside the promoters (Fig. 3A). Again, the DBPs were divided into “GAINED” and “LOST” DBPs, being 445 and 8517, respectively (Fig. 3B and C). Interestingly, as shown in Fig. 3B, the binding signal of the displaced peaks was very high in the CTRL condition whilst dramatically decreasing to zero under the ET-15d condition, suggesting a strong impact of the drug on these binding sites. In a similar way as in ET-72h, we found that the genes related to the 445 “GAINED” DBPs were not associated with any relevant biological function, while the 8517 “LOST” DBPs, associated with 4349 unique genes, were enriched in 47 significant biological pathways (Table S7, Fig. 3D). Moreover, we found the same pathways as the previous time point ET-72h, but with a far greater number of genes involved indicating an increasing restoration of the transcriptional activity, for example in the adipogenesis pathway (*N* = 54 in contrast to the previous *N* = 27) and the PI3K-Akt signaling pathway (*N *= 111 in contrast to the previous *N* = 49). Moreover, we found other biological pathways that were pivotal under control conditions, like the extracellular matrix organization, the endo- and ectoderm differentiation, and the RHO GTPase cycle. These results suggest that trabectedin may play a role in weakening FUS-DDIT3 binding to the DNA followed by the restoration the transcriptional activity of the genes previously blocked by the chimera.

### The dynamics of FUS-DDIT3 binding activity in MLPS trabectedin-resistant models during drug exposure

Previously derived RNA-Seq data from the ML017ET-resistant model shows that trabectedin is unable to induce significant changes in gene expression [15]. Whether and to what extent this drug can affect the FUS-DDIT3 binding in this model is unknown. Thus, we studied FUS-DDIT3 binding activity in ML017ET by comparing the treated conditions to the controls, *i.e.* ET-24h *vs* CTRL, ET-72h *vs* CTRL, and ET-15d *vs* CTRL, in a similar way as in the responsive ML017 model (Figure S3). For each comparison, we characterized the DBPs of FUS-DDIT3 that changed upon trabectedin treatment. The DBPs were subsequently associated with the putative target genes (Table S8).

**Fig. 4:**
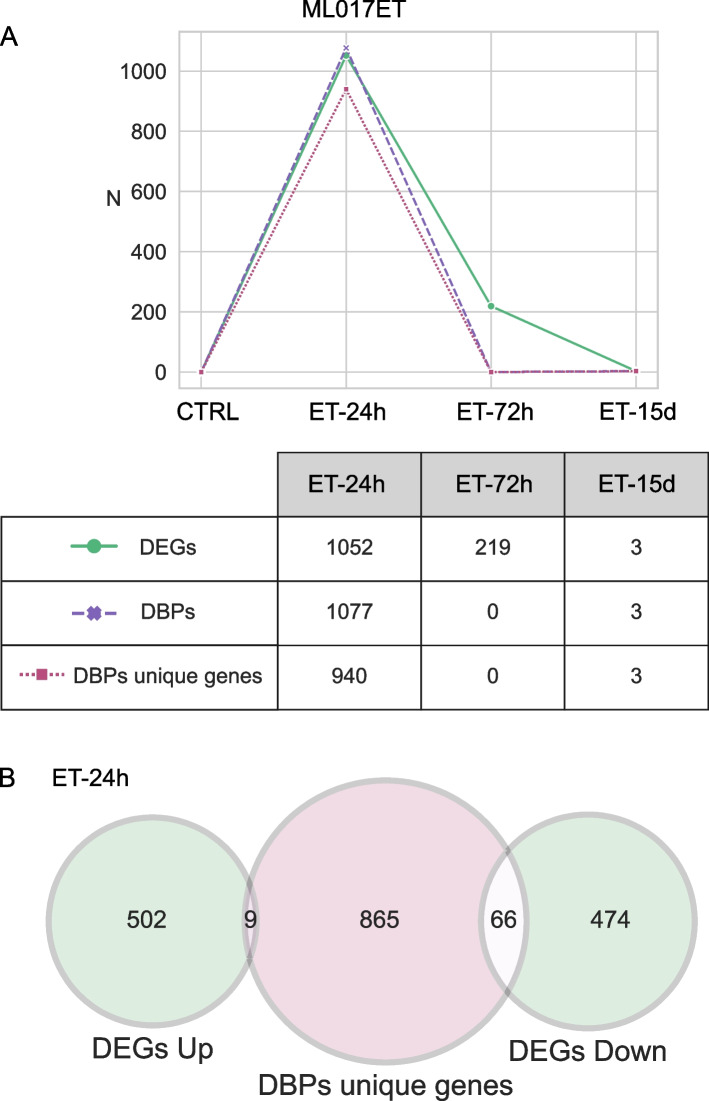
Comparison between ChIP-Seq and RNA-Seq data in ML017ET. **A** Figure shows the number (N) of differentially expressed genes (DEGs, in green), differentially bound peaks (DBPs, in pink), and unique genes associated with DBPs (in magenta), under the three trabectedin-treated conditions (ET-24h, ET-72h, ET-15d) in ML017ET model. **B** Venn diagram comparing the unique genes related to DBPs at ET-24 and the DEGs under the same condition

As shown in Fig. 4A, the total number (*N* = 1077) of DBPs at the first time point (ET-24h) rapidly decreased to zero at ET-72h maintaining this low level at ET-15d (*N* = 3). The number of unique target genes changed accordingly (940 to 0, and 3, respectively). A comparison of these results with the differentially expressed genes (DEGs) modulated by trabectedin in this model shows that both DBPs and DEGs followed the same decreasing trend over time. Indeed, 24 h after the first dose of the drug there was a slight response both in terms of DBPs (*N* = 1077) and DEGs (*N* = 1052) even though with a low overlap between genes (Fig. 4B). Prolonged treatment was not able to induce any transcription or post-transcriptional modification, thus the ML017ET model is subjected to low or no transcriptional changes and it retains the FUS-DDIT3 binding profile. Altogether, these data suggest that in ML017ET the resistance against trabectedin is mediated at transcriptional and protein levels.

### The two-phase effect of trabectedin in MLPS tumors

**Fig. 5:**
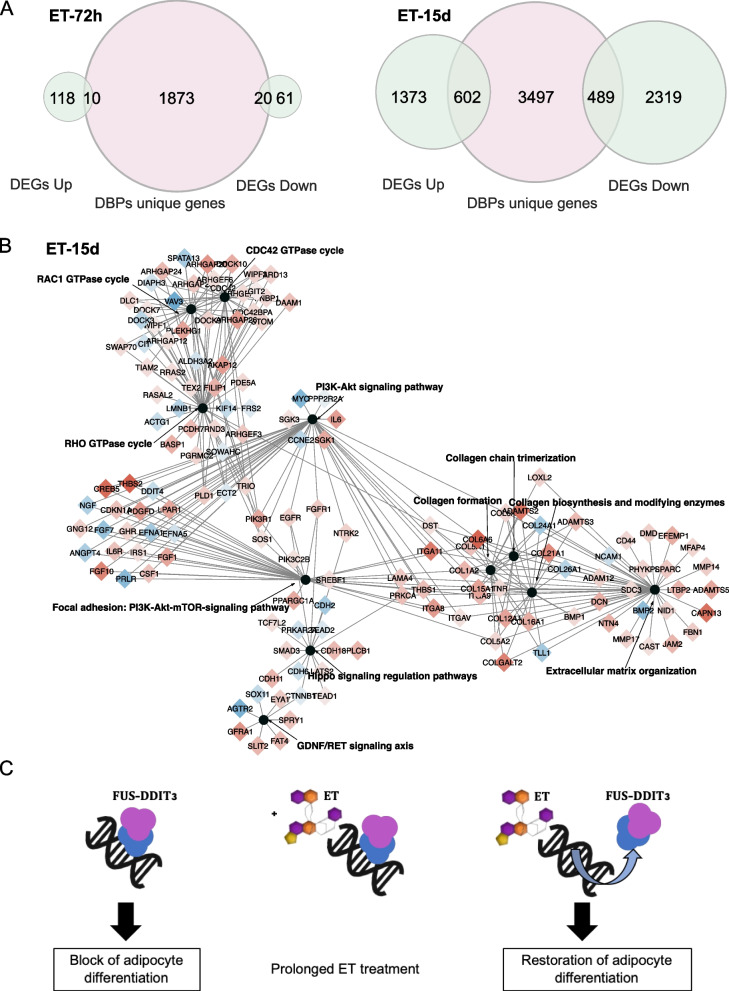
Mechanism of action of trabectedin in myxoid liposarcoma. **A** Venn diagrams showing the common genes between DEGs, divided in up- and down-regulated, and the unique genes annotated to DBPs, in ET-72h and ET-15d, respectively, in the ML017 model. **B** Enrichment map showing the common pathways between RNA-Seq and ChIP-Seq analysis at ET-15d in ML017. Common pathways are reported as black circles. Common genes are connected by an edge to the pathway they belong to, and the color shows their transcriptional regulation: red for up-regulated genes, blue for down-regulated genes. The darker the color the greater the regulation. **D** Schematic overview of the possible mechanism of action of trabectedin in myxoid liposarcoma guided by ChIP-Seq and RNA-Seq results

Once it was established that trabectedin has no effect on FUS-DDIT3 in resistant tumors, we returned to the responsive ML017 model to understand the functional role of the previously identified pathways. To this aim, we integrated ChIP-Seq and RNA-Seq data under ET-72h and ET-15d conditions. As shown in Fig. 5A, the comparison between DEGs and genes associated with DBPs showed a small number of common genes (*N* = 30) at ET-72h, while it was higher (*N* = 1091) at ET-15d. Since transcription factors mostly bind to promoter regions closer to the genes they regulate, we selected the DBPs annotated to promoters and compared the respective enriched genes to DEGs. As reported in Table S9, these results further sustain the transcriptional modulation of genes associated to the differential binding of the FUS-DDIT3 chimera at ET-15d. This was also reflected at the pathway level, where we did not find any overlap at 72 h, while a higher overlap was found at 15 days after the third dose, especially concerning the pathways related to the RHO GTPase cycle, the PI3K-Akt signaling, the Hippo pathway, extracellular matrix organization, and functions associated with the production of collagen-like collagen chain trimerization, collagen formation, and collagen biosynthesis and modifying enzymes (Fig. 5B).

As summarized in Fig. 5C, the results obtained by combining ChIP-Seq and RNA-Seq data suggest that the early effect of trabectedin is predominantly independent of FUS-DDIT3 and cytotoxic as also supported by our previous work [15]. This scenario is maintained at 72 h. At the latest time point, 15 days after the third dose, the overlap between DEGs and genes related to DBPs is the highest, suggesting that FUS-DDIT3-suppressed genes are involved in transcriptionally active pro-differentiation processes.

## Discussion

While trabectedin has demonstrated antitumor activity against various epithelial cancers such as ovarian and breast carcinomas [35, 36], as well as mesenchymal neoplasms like leiomyosarcomas and liposarcomas [4, 37], its efficacy is notably pronounced in myxoid liposarcoma (MLPS). MLPS is characterized by the expression of the FUS-DDIT3 chimeric protein, which inhibits adipocytic differentiation [12]. Remarkably durable tumor responses have been observed in responsive MLPS often manifesting as tissue-density changes preceding tumor shrinkage, suggesting a distinct mechanism of action for trabectedin [38].

Additionally, patient-derived xenografts (PDX) of MLPS have exhibited exquisite sensitivity to trabectedin, mirroring the histological and biological features of clinical tumors [20]. In these models, trabectedin’s potent antitumor activity is associated with cellular vascular depletion, along with structural changes in neoplastic tissue, resembling clinical responses [20]. Further investigations suggest that trabectedin may restore adipocytic differentiation by disrupting the transcriptional block of the FUS-DDIT3 chimera [14]. Transcriptomic data support this hypothesis, indicating trabectedin-induced transcriptional activation of genes involved in tumor morphology [15]. Previous experiments propose that trabectedin displaces FUS-DDIT3 from DNA, neutralizing its oncogenic potential [14], a specific effect not seen with doxorubicin [14].

Using ChIP-Seq, we analyzed FUS-DDIT3 binding sites in fresh tumor samples derived from MLPS PDX models for the first time. Our findings corroborate previous studies demonstrating FUS-DDIT3’s inhibitory role in adipogenesis [39]. Trabectedin modulates these binding sites, particularly after prolonged treatment, resulting in transcriptional re-activation of genes involved in differentiation (15 days after the third dose). However, prolonged trabectedin treatment in clinical settings often leads to acquired resistance. To mimic this, we exposed the drug-sensitive MLPS xenograft ML017 to trabectedin until it acquired partial resistance (ML017ET), enabling investigation of resistance [16]. Interestingly, in ML017ET, trabectedin caused transient growth delay followed by tumor regrowth without evidence of cellular depletion or adipocytic differentiation.

We do not have an explanation for the finding that trabectedin affects the DNA binding of FUS-DDIT3 in the sensitive ML017 tumor but not in the resistant ML017ET tumor. Since the DNA binding of FUS-DDIT3 was found to be similar in ML017 and in ML017ET tumors, it may be speculated that the detachment caused by trabectedin involves some other still unidentified factors. In a previous study we found that the ML017ET model is deficient in UV stimulated scaffold protein (UVSSA) [15] that plays a role in the transcription-coupled nucleotide excision repair (TC-NER) pathway to help remove lesions in the DNA that block transcription. Therefore we can speculate that UVSSA enhances the ability of trabectedin to detach FUS-DDIT3 from DNA in ML017 tumor and its absence in ML017ET tumor limits this mechanism. This hypothesis is entirely speculative and requires experimental evidence.

Unable to conduct detailed mechanistic studies in MLPS patients, we utilized preclinical models to elucidate trabectedin’s selective mode of action and mechanisms of acquired resistance, likely similar to those in patients. Notably, our studies utilized therapeutic i.v. doses and the morphological changes observed in ML017 closely resembled those in human samples [20].

## Conclusions

In summary, we revealed trabectedin’s unique mechanism in MLPS by inhibiting the FUS-DDIT3 binding of target genes, removing the differentiation block. Resistance in ML017ET is associated with loss of trabectedin’s ability to induce adipocytic differentiation and changes in tumor tissue morphology. This observation’s clinical relevance is supported by the absence of radiological tissue changes in trabectedin-unresponsive patients [38]. A potential strategy to counter resistance involves combining trabectedin with the PPAR agonist pioglitazone, as suggested by Frapolli et al*.* [40]. Clinical trials (EudraCT Number: 2020–005626-29) are underway to evaluate this approach in MLPS patients.

## Supplementary Information

Additional file 1: Figure S1. Results of the motif analysis run on Pscan-ChIP (24) on the consensus peaks from the CTRL condition of ML017. The figure shows A) the information on the most represented motif DDIT3 (MA0019.1, as reported in the Jaspar database (25)), B) the associated matrix, C) the sequence logo, and D) the positions of the best occurrences. Figure S2. Genomic distribution (%) of the annotated differentially bound peaks from the comparison between ML017ET CTRL and ML017 CTRL. Color regions as reported in the legend. Figure S3. Schema of the conditions used in this work: CTRL, control for basal conditions; ET-24h and ET-72h, 24 and 72 hours after the first dose of trabectedin (ET), respectively, for early effects analysis; ET-15d, 15 days after the third dose of ET for late effects analysis. Figure S4. Figure shows the number (N) of differentially expressed genes (DEGs, in green), differentially bound peaks (DBPs, in violet), and unique genes associated with DBPs (in magenta), in the ML017 model under the three different conditions, ET-24h, ET-72h, and ET-15d. Figure S5. A) Venn diagrams showing the common genes between DEGs, divided in up- and down-regulated, and the unique genes annotated to DBPs, in ET-72h and ET-15d, respectively, in the ML017 model. B) Enrichment map showing the common pathways between RNA-Seq and ChIP-Seq analysis at ET-15d in ML017. Common pathways are reported as black circles. Common genes are connected by an edge to the pathway they belong to, and the color shows their transcriptional regulation: red for up-regulated genes, blue for down-regulated genes. The darker the color the greater the regulation. Table S3. Results from the Pscan-ChIP [26] analysis on the consensus peaks from the CTRL condition of ML017. Table reports the first five most represented motives in the analyzed consensus. Motif Name, the name of the identified motif; Motif ID, identifier of the motif as reported in the Jaspar 2018 database [27]; Global *p*-value, statistical significance of the motif; Position, position of the identified motif in the analyzed peaks. Table S9. Table shows the number of differentially bound peaks (DBPs) that have been annotated to promoter regions under each condition (ET-24h, ET-72h, and ET-15d) and further divided into gained or lost regions according to the fold change of the DBPs analysis. The number of associated enriched genes that are also transcriptionally modulated according to RNA-Seq analysis are indicated.

Additional file 2: Table S1. Table reports the samples analyzed in this work. SampleID, the name of the sample; PDX model, patient-derived xenograft model of origin; Condition, kind of condition related to the sample: Treatment, kind of treatment, i.e. trabectedin or doxorubicin or none; Replicate, replicate number: ControlID, identifier of the input sample used for the analysis. See attached excel file.

Additional file 3: Table S2. Annotated consensus peaks derived from the control samples of the ML017 model. Chrom, chromosome; start and end indicate the starting and the ending position of the consensus peak; width indicate the extension of the consensus peak in bp; annotation indicates the genomic region associated with the consensus peak; geneStart and geneEnd represent the starting and the ending position of the annotated gene; geneLength is the length of the annotated gene; geneStrand represents the reading strand of the annotated gene, 1 for forward, 2 for reverse; geneID is the official Entrez identifier of the annotated gene; distanceToTSS indicates the distance in bp from the transcription start site (TSS) of the annotated gene; ENSEMBL is the official ENSEMBL identifier associated with the annotated gene; SYMBOL is the official HUGO gene symbol identifier for the annotated gene. See attached excel file.

Additional file 4: Table S4. Pathway enrichment results from the consensus of the control peaks in ML017. ID, the identifier of the pathway as stored in the database of origin; Description, the extended name of the pathway; *p*-value, statistical *p*-value; q-value, multiple testing corrected *p*-value; Count, number of genes in pathways. See attached excel file.

Additional file 5: Table S5. Differentially bound peaks (DBPs) from the comparison between ML017ET control (CTRL) conditions and ML017 CTRL. Chrom, chromosome; start and end indicate the starting and the ending position of the consensus peak; width indicate the extension of the consensus peak in bp; Conc is the mean read concentration over all the samples, i.e. log2 normalized ChIP read counts with read of the reference control sample subtracted; Conc_ML017ET_ctrl, mean read concentration over the ML017ET at basal conditions; Conc_ML017_ctrl, mean read concentration over the ML017 at basal conditions; Fold represents the differences in mean concentration between the compared groups; *p*.value is the significance of the comparison; FDR is the false discovery rate corrected *p*-value; annotation indicates the genomic region associated with the consensus peak; geneStart and geneEnd represent the starting and the ending position of the annotated gene; geneLength is the length of the annotated gene; geneStrand represents the reading strand of the annotated gene, 1 for forward, 2 for reverse; geneID is the official Entrez identifier of the annotated gene; distanceToTSS indicates the distance in bp from the transcription start site (TSS) of the annotated gene; ENSEMBL is the official ENSEMBL identifier associated with the annotated gene; SYMBOL is the official HUGO gene symbol identifier for the annotated gene; GENENAME is the official gene name of the annotated gene. See attached excel file.

Additional file 6: Table S6. Differentially bound peaks (DBPs) of the ML017 model from the comparison ET-24h vs CTRL (sheet name reported as ET-24 vs CTRL), ET-72h vs CTRL (sheet name reported as ET-72 vs CTRL), and ET-15d vs CTRL (sheet name reported as ET-15 vs CTRL). For each sheet name columns legend is as follows: Chrom, chromosome; start and end indicate the starting and the ending position of the consensus peak; width indicate the extension of the consensus peak in bp; Conc is the mean read concentration over all the samples, i.e. log2 normalized ChIP read counts with read of the reference control sample subtracted; Conc_ML017_ET-24/ET-72/ET-15, mean read concentration over the selected condition; Conc_ML017_ctrl, mean read concentration over the ML017 at basal conditions; Fold represents the differences in mean concentration between the compared groups; *p*.value is the significance of the comparison; FDR is the false discovery rate corrected p-value; annotation indicates the genomic region associated with the consensus peak; geneStart and geneEnd represent the starting and the ending position of the annotated gene; geneLength is the length of the annotated gene; geneStrand represents the reading strand of the annotated gene, 1 for forward, 2 for reverse; geneID is the official Entrez identifier of the annotated gene; distanceToTSS indicates the distance in bp from the transcription start site (TSS) of the annotated gene; ENSEMBL is the official ENSEMBL identifier associated with the annotated gene; SYMBOL is the official HUGO gene symbol identifier for the annotated gene; GENENAME is the official gene name of the annotated gene. See attached excel file.

Additional file 7: Table S7. Pathway enrichment results from the ET-15d condition in ML017. ID, the identifier of the pathway as stored in the database of origin; Description, the extended name of the pathway; *p*-value, statistical *p*-value; q-value, multiple testing corrected *p*-value; Count, number of genes in pathways. See attached excel file.

Additional file 8: Table S8. Differentially bound peaks (DBPs) of the ML017ET model from the comparison ET-24h vs CTRL (sheet name reported as ET-24 vs CTRL), and ET-15d vs CTRL (sheet name reported as ET-15 vs CTRL). For each sheet name columns legend is as follows: Chrom, chromosome; start and end indicate the starting and the ending position of the consensus peak; width indicate the extension of the consensus peak in bp; Conc is the mean read concentration over all the samples, i.e. log2 normalized ChIP read counts with read of the reference control sample subtracted; Conc_ML017ET-24/ET-15, mean read concentration over the selected condition; Conc_ML017ET_ctrl, mean read concentration over the ML017 at basal conditions; Fold represents the differences in mean concentration between the compared groups; *p*.value is the significance of the comparison; FDR is the false discovery rate corrected *p*-value; annotation indicates the genomic region associated with the consensus peak; geneStart and geneEnd represent the starting and the ending position of the annotated gene; geneLength is the length of the annotated gene; geneStrand represents the reading strand of the annotated gene, 1 for forward, 2 for reverse; geneID is the official Entrez identifier of the annotated gene; distanceToTSS indicates the distance in bp from the transcription start site (TSS) of the annotated gene; ENSEMBL is the official ENSEMBL identifier associated with the annotated gene; SYMBOL is the official HUGO gene symbol identifier for the annotated gene; GENENAME is the official gene name of the annotated gene. See attached excel file.

## Data Availability

The datasets generated and analyzed during the current study are available in the European Genome-phenome Archive (EGA) repository with accession number EGAD00001005099 (https://ega-archive.org/search/EGAD00001005099), and in the European Nucleotide Archive (ENA) at EMBL-EBI under accession number PRJEB74484 (https://www.ebi.ac.uk/ena/browser/view/PRJEB74484).

## Abbreviations

DBPs: Differentially bound peaks
DEGs: Differentially expressed genes
DOXO: Doxorubicin
EMA: European Medicine Agency
ET: Trabectedin
FDA: Food and Drug Administration
MLPS: Myxoid liposarcoma
PDX: Patient-derived xenograft
TC-NER: Transcription-coupled nucleotide excision repair
TSS: Transcription start site
