# Supplementary material for: Mechanism of efficacy of trabectedin against myxoid liposarcoma entails detachment of the FUS-DDIT3 transcription factor from its DNA binding sites
Source: J Exp Clin Cancer Res. 2024 Nov 26;43:309. doi: 10.1186/s13046-024-03228-z (PMC11590625; doi:10.1186/s13046-024-03228-z)
Supplement: Supplementary file 1 — Additional file 1: Figure S1. Results of the motif analysis run on Pscan-ChIP (24) on the consensus peaks from the CTRL condition of ML017. The figure shows A) the information on the most represented motif DDIT3 (MA0019.1, as reported in the Jaspar database (25)), B) the associated matrix, C) the sequence logo, and D) the positions of the best occurrences. Figure S2. Genomic distribution (%) of the annotated differentially bound peaks from the comparison between ML017ET CTRL and ML017 CTRL. Color regions as reported in the legend. Figure S3. Schema of the conditions used in this work: CTRL, control for basal conditions; ET-24h and ET-72h, 24 and 72 hours after the first dose of trabectedin (ET), respectively, for early effects analysis; ET-15d, 15 days after the third dose of ET for late effects analysis. Figure S4. Figure shows the number (N) of differentially expressed genes (DEGs, in green), differentially bound peaks (DBPs, in violet), and unique genes associated with DBPs (in magenta), in the ML017 model under the three different conditions, ET-24h, ET-72h, and ET-15d. Figure S5. A) Venn diagrams showing the common genes between DEGs, divided in up- and down-regulated, and the unique genes annotated to DBPs, in ET-72h and ET-15d, respectively, in the ML017 model. B) Enrichment map showing the common pathways between RNA-Seq and ChIP-Seq analysis at ET-15d in ML017. Common pathways are reported as black circles. Common genes are connected by an edge to the pathway they belong to, and the color shows their transcriptional regulation: red for up-regulated genes, blue for down-regulated genes. The darker the color the greater the regulation. Table S3. Results from the Pscan-ChIP [26] analysis on the consensus peaks from the CTRL condition of ML017. Table reports the first five most represented motives in the analyzed consensus. Motif Name, the name of the identified motif; Motif ID, identifier of the motif as reported in the Jaspar 2018 database [27]; Global p-valu [file 13046_2024_3228_MOESM1_ESM.docx]

# Supplementary data to

# Mechanism of efficacy of trabectedin against myxoid liposarcoma entails detachment of the FUS-DDIT3 transcription factor from its DNA binding sites

Ilaria Craparotta^1*^, Laura Mannarino^2,3*^, Riccardo Zadro^2,3*^, Sara Ballabio^4^, Sergio Marchini^3^, Giulio Pavesi^5^, Marta Russo^6^, Salvatore Lorenzo Renne^2,7^, Marina Meroni^1^, Marianna Ponzo^1^, Ezia Bello^1^, Roberta Sanfilippo^8^, Paolo G. Casali^8^, Maurizio D’Incalci^2,3†^, Roberta Frapolli^1^

**Affiliations**

^1^Department of Oncology, Istituto di Ricerche Farmacologiche Mario Negri IRCCS, Milan, Italy

^2^Department of Biomedical Sciences, Humanitas University, via Rita Levi Montalcini 4, 20072 Pieve Emanuele, Milan, Italy

^3^Laboratory of Cancer Pharmacology, IRCCS Humanitas Research Hospital, via Manzoni 56, 20089 Rozzano, Milan, Italy

^4^SC Patologia Clinica, SS Laboratorio Genetica Medica, Fondazione IRCCS Ca’ Granda Ospedale Maggiore Policlinico, Milan, Italy

^5^Dipartimento di Bioscienze, Università degli Studi di Milano, 20133, Milan, Italy

^6^Department of Experimental Oncology, European Institute of Oncology (IEO) IRCCS, Milan 20139, Italy

^7^Anatomic Pathology Unit, IRCCS Humanitas Research Hospital, via Manzoni 56, 20089 Rozzano, Milan, Italy

^8^Adult Mesenchymal Tumour Medical Oncology Unit, Fondazione IRCCS Istituto Nazionale dei Tumori, Via Venezian 1, 20133 Milan, Italy

# Supplementary Figures


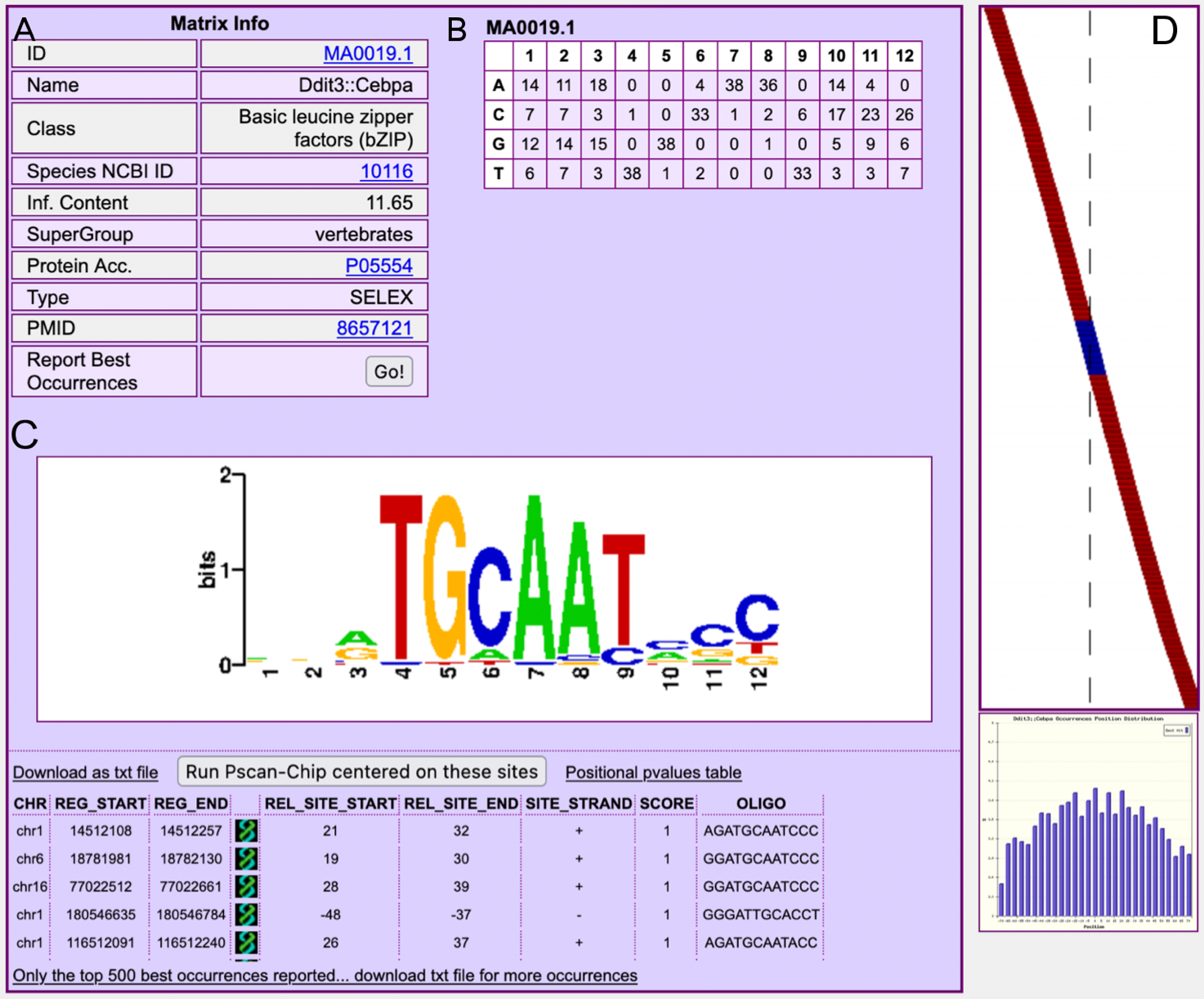


**Figure S1.** Results of the motif analysis run on Pscan-ChIP (24) on the consensus peaks from the CTRL condition of ML017. The figure shows A) the information on the most represented motif DDIT3 (MA0019.1, as reported in the Jaspar database (25)), B) the associated matrix, C) the sequence logo, and D) the positions of the best occurrences.


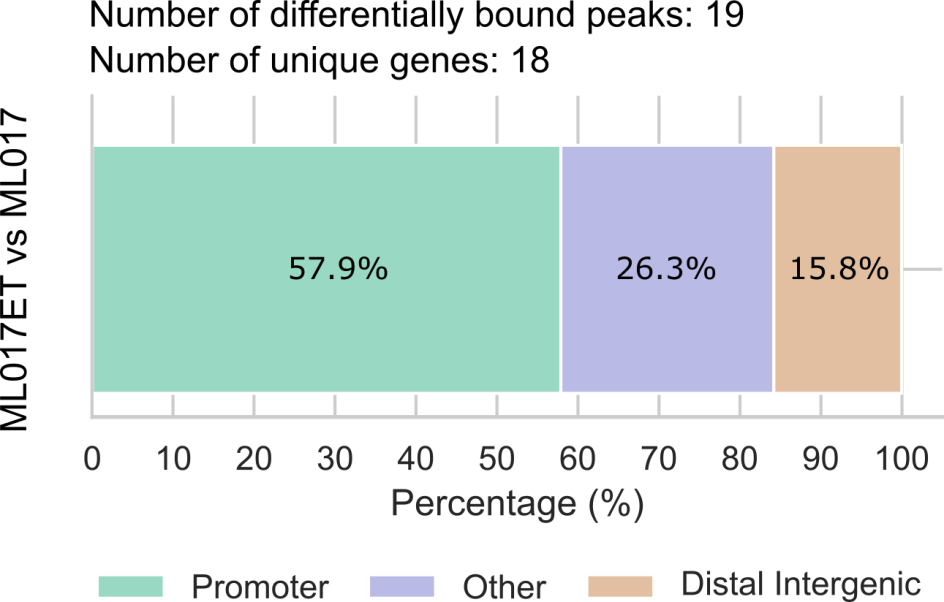


**Figure S2.** Genomic distribution (%) of the annotated differentially bound peaks from the comparison between ML017ET CTRL and ML017 CTRL. Color regions as reported in the legend.


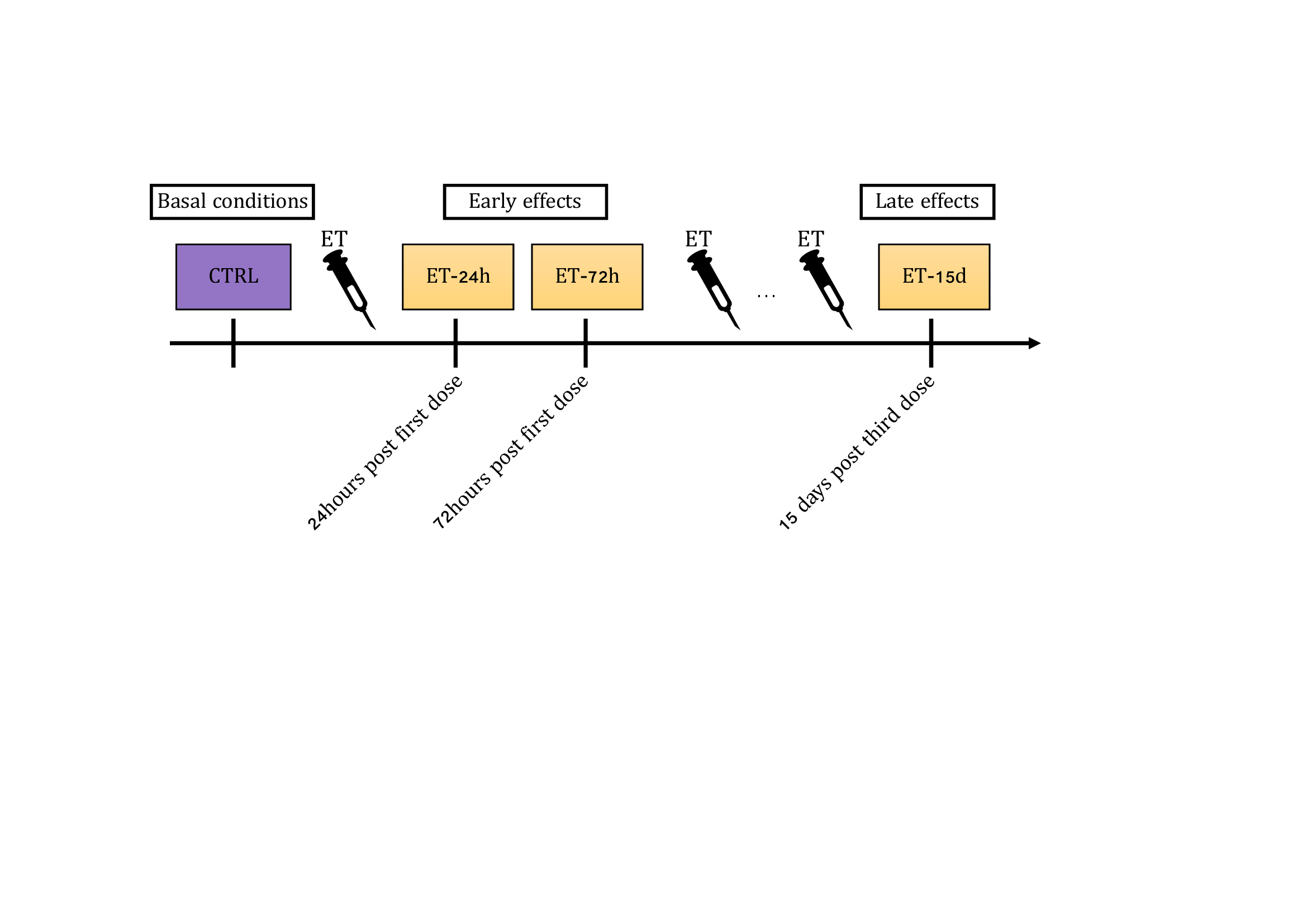


**Figure S3.** Schema of the conditions used in this work: CTRL, control for basal conditions; ET-24h and ET-72h, 24 and 72 hours after the first dose of trabectedin (ET), respectively, for early effects analysis; ET-15d, 15 days after the third dose of ET for late effects analysis.

**
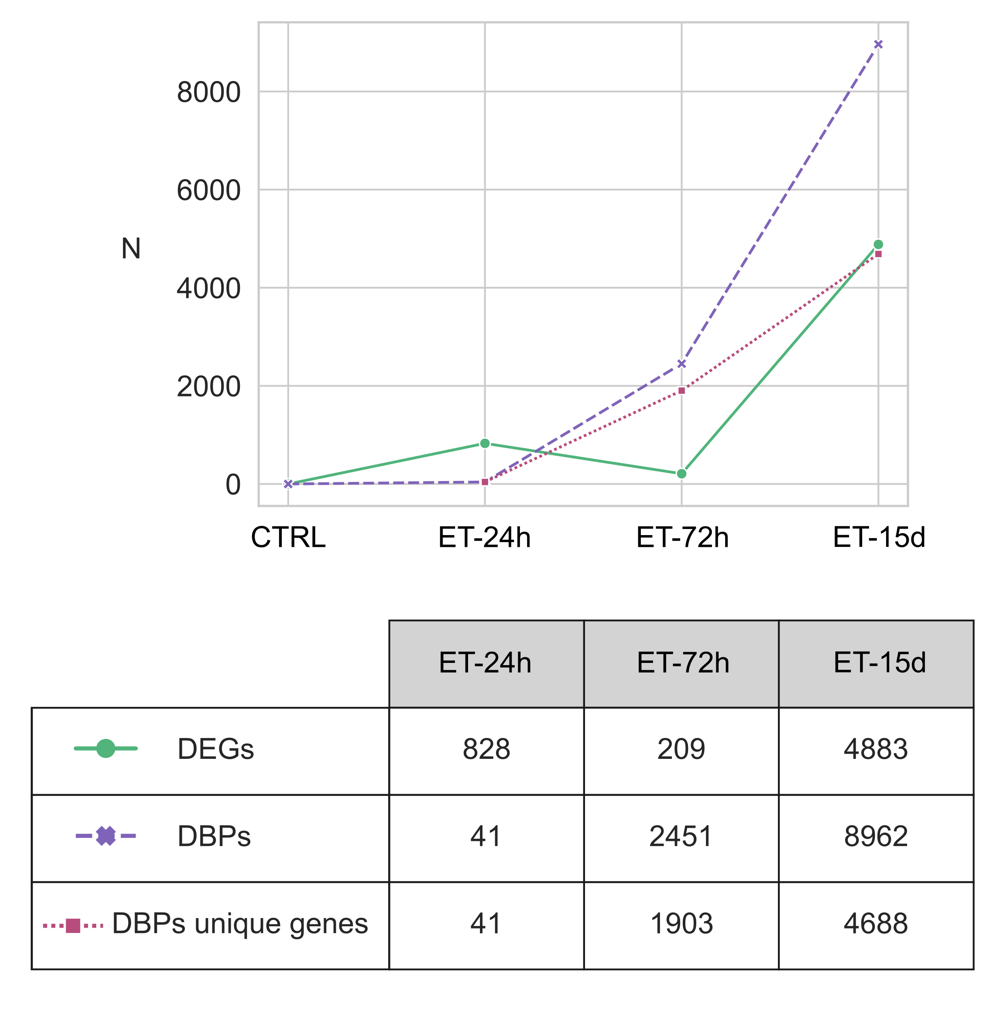
**

**Figure S4.** Figure shows the number (N) of differentially expressed genes (DEGs, in green), differentially bound peaks (DBPs, in violet), and unique genes associated with DBPs (in magenta), in the ML017 model under the three different conditions, ET-24h, ET-72h, and ET-15d.

# Supplementary Tables

**Table S3**

| **Motif Name** | **Motif ID (JASPAR2018)** | **Global p-value** | **Position** |
| --- | --- | --- | --- |
| Ddit3::Cebpa | MA0019.1 | < 0.001 - Over | [-4,6] |
| ATF4 | MA0833.1 | < 0.001 - Over | [1,11] |
| CEBPA | MA0102.3 | < 0.001 - Over | [-10,0] |
| CEBPD | MA0836.1 | < 0.001 - Over | [-10,0] |
| CEBPE | MA0837.1 | < 0.001 - Over | [-10,0] |

Results from the Pscan-ChIP (26) analysis on the consensus peaks from the CTRL condition of ML017. Table reports the first five most represented motives in the analyzed consensus. Motif Name, the name of the identified motif; Motif ID, identifier of the motif as reported in the Jaspar 2018 database (27); Global p-value, statistical significance of the motif; Position, position of the identified motif in the analyzed peaks.

**Table S9**

|  | **ET-24h** | | **ET-72h** | | **ET-15d** | |
| --- | --- | --- | --- | --- | --- | --- |
| **Number of DBPs in promoter regions** | 35 | | 295 | | 925 | |
|  | **Gained** | **Lost** | **Gained** | **Lost** | **Gained** | **Lost** |
|  | 35 | 0 | 68 | 227 | 417 | 508 |
| **Enriched genes** | 35 | 0 | 68 | 223 | 415 | 482 |
| **Enriched genes that are transcriptionally modulated** | 3 | 0 | 1 | 6 | 144 | 122 |

Table shows the number of differentially bound peaks (DBPs) that have been annotated to promoter regions under each condition (ET-24h, ET-72h, and ET-15d) and further divided into gained or lost regions according to the fold change of the DBPs analysis. The number of associated enriched genes that are also transcriptionally modulated according to RNA-Seq analysis are indicated.
